# 3-(1,3-Dithio­lan-2-yl­idene)-1-(4-meth­oxy­phen­yl)pyridine-2,4(1*H*,3*H*)-dione

**DOI:** 10.1107/S1600536811033009

**Published:** 2011-08-27

**Authors:** Yan-Chun Ma, Jin-Long Song

**Affiliations:** a Educational Institute of Jilin Province, Changchun 130022, People’s Republic of China; b Jinlin Province Product Quality Supervision Test Institute, Changchun 130022, People’s Republic of China

## Abstract

In the title compound, C_15_H_13_NO_3_S_2_, the dithiol­ane ring adopts a twisted conformation. The mol­ecule exhibits a V-shaped conformation, with a dihedral angle of 79.05 (7)° between the benzene ring and the pyridine ring. In the crystal, C—H⋯O inter­actions are observed.

## Related literature

For the synthesis, see: Li *et al.* (2008 ▶). For background to *N*-substituted pyridine compounds and their potential use in medicinal chemistry, see: Kim *et al.* (2008 ▶); Zhu *et al.* (2006 ▶)
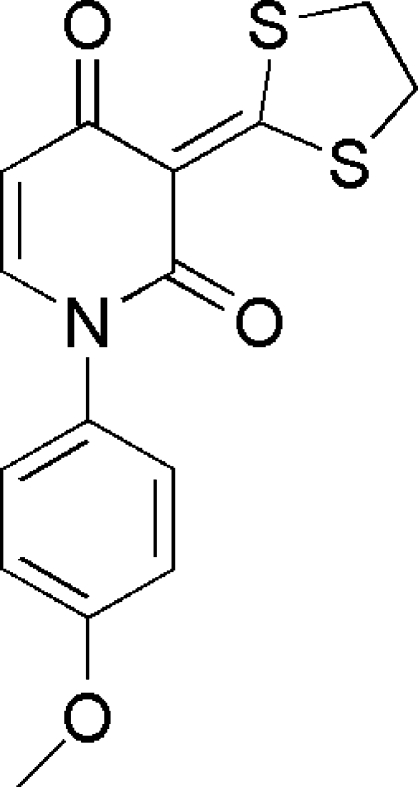


## Experimental

### 

#### Crystal data

C_15_H_13_NO_3_S_2_
*M* *_r_* = 319.40
Monoclinic, 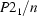

*a* = 5.322 (2) Å
*b* = 27.521 (11) Å
*c* = 10.065 (4) Å
β = 100.831 (5)°
*V* = 1448.0 (10) Å^3^
*Z* = 4
Mo *K*α radiation
μ = 0.38 mm^−1^
*T* = 293 K
0.35 × 0.29 × 0.28 mm

#### Data collection

Bruker APEXII CCD diffractometer
Absorption correction: multi-scan (*SADABS*; Bruker, 2002 ▶) *T*_min_ = 0.892, *T*_max_ = 0.912
12346 measured reflections
2905 independent reflections
1973 reflections with *I* > 2σ(*I*)
*R*_int_ = 0.053

#### Refinement

*R*[*F* ^2^ > 2σ(*F* ^2^)] = 0.045
*wR*(*F* ^2^) = 0.121
*S* = 1.08
2905 reflections
190 parameters
H-atom parameters constrained
Δρ_max_ = 0.43 e Å^−3^
Δρ_min_ = −0.30 e Å^−3^

### 

Data collection: *APEX2* (Bruker, 2002 ▶); cell refinement: *SAINT* (Bruker, 2002 ▶); data reduction: *SAINT*; program(s) used to solve structure: *SHELXS97* (Sheldrick, 2008 ▶); program(s) used to refine structure: *SHELXL97* (Sheldrick, 2008 ▶); molecular graphics: *SHELXTL* (Sheldrick, 2008 ▶); software used to prepare material for publication: *SHELXTL*.

## Supplementary Material

Crystal structure: contains datablock(s) global, I. DOI: 10.1107/S1600536811033009/ff2024sup1.cif

Structure factors: contains datablock(s) I. DOI: 10.1107/S1600536811033009/ff2024Isup2.hkl

Supplementary material file. DOI: 10.1107/S1600536811033009/ff2024Isup3.cml

Additional supplementary materials:  crystallographic information; 3D view; checkCIF report

## Figures and Tables

**Table 1:** Hydrogen-bond geometry (Å, °)

*D*—H⋯*A*	*D*—H	H⋯*A*	*D*⋯*A*	*D*—H⋯*A*
C8—H8⋯O2^i^	0.93	2.42	3.259 (3)	150

Symmetry code: (i) 
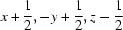
.

**Fig. 1.**
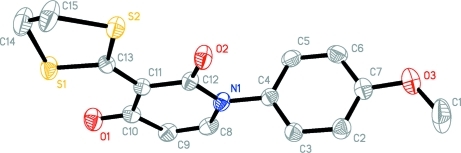
Molecular structure of the title compound with displacement ellipsoids drawn at the 30% probability level for non-H atoms.

## supplementary crystallographic information

### Comment

Among the richness of heterocyclic compounds, N-substituted pyridone compounds (Zhu *et al.*, 2006) have attracted an intense interest due to their potential for medicinal chemistry (Kim *et al.*, 2008). Recently, a large number of N-substituted pyridone compounds have been prepared (Li *et al.*, 2008). As a contribution to this field, the structure of the title crystal is presented here. The molecular structure of the title compound, together with the atom-numbering scheme, is illustrated in Fig.1. Selected bond lengths and angles are given in Table 1. The molecule exhibits a V-shaped conformation in the crystal with a dihedral angle of 79.05 (7)° between the benzene ring and the pyridine ring. The dithiolane ring has a twisted conformation.

### Experimental

The title compound was synthesized according to the literature (Li *et al.*, 2008). It was dissolved in ethyl acetate at room temperature and hexane was added. The solution was kept at room temperature in a sealed flask for a few days to give single crystals suitable for single-crystal X-ray analysis.

### Refinement

All H atoms bound to C atoms were generated geometrically and refined as riding atoms with C—H= 0.93Å for aromatic H , 0.96Å for CH_3_ groups, 0.97Å for CH_2_ groups, and with *U*_iso_(H) = 1.5*U*_eq_(C) for CH_3_ groups and *U*_iso_(H) = 1.2*U*_eq_(C) for all the other groups.

### Crystal data

**Table:**

C_15_H_13_NO_3_S_2_	*Z* = 4
*M**_r_* = 319.40	*F*(000) = 664
Monoclinic, *P*2_1_/*n*	*D*_x_ = 1.453 Mg m^−^^3^
Hall symbol: -p 2yn	Mo *K*α radiation, λ = 0.71073 Å
*a* = 5.322 (2) Å	θ = 2.7–27.1°
*b* = 27.521 (11) Å	µ = 0.38 mm^−^^1^
*c* = 10.065 (4) Å	*T* = 293 K
β = 100.831 (5)°	Block, yellow
*V* = 1448.0 (10) Å^3^	0.35 × 0.29 × 0.28 mm

### Data collection

**Table:**

Bruker APEXII CCD diffractometer	2905 independent reflections
Radiation source: fine-focus sealed tube	1973 reflections with *I* > 2σ(*I*)
graphite	*R*_int_ = 0.053
ω scans	θ_max_ = 26.2°, θ_min_ = 2.2°
Absorption correction: multi-scan (*SADABS*; Bruker, 2002)	*h* = −6→6
*T*_min_ = 0.892, *T*_max_ = 0.912	*k* = −34→33
12346 measured reflections	*l* = −12→12

### Refinement

**Table:**

Refinement on *F*^2^	Primary atom site location: structure-invariant direct methods
Least-squares matrix: full	Secondary atom site location: difference Fourier map
*R*[*F*^2^ > 2σ(*F*^2^)] = 0.045	Hydrogen site location: inferred from neighbouring sites
*wR*(*F*^2^) = 0.121	H-atom parameters constrained
*S* = 1.08	*w* = 1/[σ^2^(*F*_o_^2^) + (0.0581*P*)^2^] where *P* = (*F*_o_^2^ + 2*F*_c_^2^)/3
2905 reflections	(Δ/σ)_max_ < 0.001
190 parameters	Δρ_max_ = 0.43 e Å^−^^3^
0 restraints	Δρ_min_ = −0.30 e Å^−^^3^

### Special details

**Table:**

Geometry. All esds (except the esd in the dihedral angle between two l.s. planes) are estimated using the full covariance matrix. The cell esds are taken into account individually in the estimation of esds in distances, angles and torsion angles; correlations between esds in cell parameters are only used when they are defined by crystal symmetry. An approximate (isotropic) treatment of cell esds is used for estimating esds involving l.s. planes.
Refinement. Refinement of F^2^ against ALL reflections. The weighted R-factor wR and goodness of fit S are based on F^2^, conventional R-factors R are based on F, with F set to zero for negative F^2^. The threshold expression of F^2^ > 2sigma(F^2^) is used only for calculating R-factors(gt) etc. and is not relevant to the choice of reflections for refinement. R-factors based on F^2^ are statistically about twice as large as those based on F, and R- factors based on ALL data will be even larger.

### Fractional atomic coordinates and isotropic or equivalent isotropic displacement parameters (Å^2^)

**Table:**

	*x*	*y*	*z*	*U*_iso_*/*U*_eq_	
S2	0.24453 (13)	0.13081 (2)	1.20138 (7)	0.0510 (2)	
S1	0.45027 (15)	0.05404 (2)	1.05151 (8)	0.0620 (3)	
N1	0.6394 (4)	0.23410 (7)	0.9727 (2)	0.0462 (5)	
O2	0.3943 (3)	0.21607 (6)	1.12752 (18)	0.0595 (5)	
O1	0.7470 (4)	0.09067 (7)	0.89857 (19)	0.0670 (5)	
O3	0.5220 (4)	0.43233 (7)	1.0589 (2)	0.0736 (6)	
C13	0.4297 (4)	0.11562 (9)	1.0831 (2)	0.0418 (6)	
C11	0.5545 (4)	0.14965 (8)	1.0179 (2)	0.0419 (6)	
C4	0.6118 (5)	0.28548 (8)	0.9964 (2)	0.0452 (6)	
C12	0.5218 (4)	0.20108 (8)	1.0456 (2)	0.0436 (6)	
C5	0.3930 (5)	0.30960 (10)	0.9369 (3)	0.0586 (7)	
H5	0.2618	0.2929	0.8811	0.070*	
C6	0.3694 (5)	0.35832 (10)	0.9602 (3)	0.0629 (8)	
H6	0.2219	0.3747	0.9197	0.075*	
C3	0.8034 (5)	0.31005 (9)	1.0787 (2)	0.0516 (6)	
H3	0.9504	0.2936	1.1193	0.062*	
C7	0.5642 (5)	0.38348 (9)	1.0438 (3)	0.0528 (6)	
C10	0.7144 (5)	0.13424 (10)	0.9219 (2)	0.0484 (6)	
C8	0.7884 (5)	0.21885 (10)	0.8818 (2)	0.0522 (7)	
H8	0.8649	0.2423	0.8362	0.063*	
C9	0.8276 (5)	0.17249 (10)	0.8563 (3)	0.0525 (6)	
H9	0.9309	0.1646	0.7944	0.063*	
C2	0.7809 (5)	0.35902 (9)	1.1022 (3)	0.0566 (7)	
H2	0.9130	0.3755	1.1578	0.068*	
C14	0.2073 (6)	0.03558 (11)	1.1426 (4)	0.0849 (10)	
H14A	0.0436	0.0341	1.0811	0.102*	
H14B	0.2466	0.0034	1.1800	0.102*	
C15	0.1914 (7)	0.06899 (10)	1.2493 (4)	0.0856 (11)	
H15A	0.3178	0.0604	1.3284	0.103*	
H15B	0.0236	0.0665	1.2733	0.103*	
C1	0.7075 (8)	0.45886 (12)	1.1489 (4)	0.1129 (14)	
H1A	0.6552	0.4922	1.1501	0.169*	
H1B	0.8688	0.4570	1.1197	0.169*	
H1C	0.7246	0.4454	1.2382	0.169*	

### Atomic displacement parameters (Å^2^)

**Table:**

	*U*^11^	*U*^22^	*U*^33^	*U*^12^	*U*^13^	*U*^23^
S2	0.0644 (4)	0.0431 (4)	0.0516 (4)	−0.0063 (3)	0.0267 (3)	−0.0052 (3)
S1	0.0783 (5)	0.0396 (4)	0.0762 (5)	0.0019 (3)	0.0355 (4)	−0.0043 (3)
N1	0.0534 (12)	0.0425 (12)	0.0455 (12)	−0.0073 (9)	0.0167 (10)	−0.0016 (9)
O2	0.0830 (13)	0.0423 (10)	0.0643 (12)	−0.0049 (9)	0.0424 (11)	−0.0071 (8)
O1	0.0835 (14)	0.0528 (12)	0.0735 (13)	0.0071 (10)	0.0375 (11)	−0.0083 (10)
O3	0.0977 (16)	0.0497 (12)	0.0752 (14)	0.0139 (11)	0.0208 (12)	0.0030 (10)
C13	0.0456 (14)	0.0412 (13)	0.0390 (13)	0.0025 (10)	0.0093 (10)	−0.0032 (10)
C11	0.0436 (13)	0.0427 (13)	0.0409 (14)	0.0002 (11)	0.0112 (11)	−0.0011 (11)
C4	0.0471 (14)	0.0448 (15)	0.0462 (14)	−0.0043 (11)	0.0152 (11)	0.0055 (11)
C12	0.0495 (14)	0.0439 (14)	0.0389 (13)	−0.0055 (11)	0.0126 (11)	−0.0008 (11)
C5	0.0442 (15)	0.0590 (18)	0.0696 (18)	−0.0090 (13)	0.0031 (13)	0.0087 (14)
C6	0.0475 (16)	0.0624 (18)	0.079 (2)	0.0068 (14)	0.0109 (14)	0.0181 (16)
C3	0.0512 (15)	0.0476 (16)	0.0530 (16)	0.0020 (12)	0.0020 (12)	0.0010 (12)
C7	0.0675 (18)	0.0476 (15)	0.0478 (15)	0.0033 (14)	0.0220 (13)	0.0053 (12)
C10	0.0489 (15)	0.0541 (17)	0.0440 (15)	0.0022 (12)	0.0136 (11)	−0.0026 (12)
C8	0.0502 (15)	0.0654 (18)	0.0446 (15)	−0.0103 (13)	0.0179 (12)	0.0012 (13)
C9	0.0538 (15)	0.0616 (17)	0.0471 (15)	−0.0036 (13)	0.0224 (12)	−0.0070 (13)
C2	0.0629 (17)	0.0517 (17)	0.0518 (16)	−0.0027 (13)	0.0017 (13)	−0.0031 (13)
C14	0.111 (3)	0.0483 (17)	0.110 (3)	−0.0121 (17)	0.058 (2)	−0.0059 (18)
C15	0.126 (3)	0.0514 (18)	0.096 (2)	−0.0232 (18)	0.065 (2)	−0.0066 (17)
C1	0.166 (4)	0.060 (2)	0.101 (3)	0.015 (2)	−0.005 (3)	−0.026 (2)

### Geometric parameters (Å, °)

**Table:**

S2—C13	1.733 (2)	C6—C7	1.391 (4)
S2—C15	1.805 (3)	C6—H6	0.9300
S1—C13	1.732 (3)	C3—C2	1.377 (3)
S1—C14	1.793 (3)	C3—H3	0.9300
N1—C8	1.384 (3)	C7—C2	1.369 (3)
N1—C12	1.389 (3)	C10—C9	1.434 (3)
N1—C4	1.446 (3)	C8—C9	1.326 (3)
O2—C12	1.233 (3)	C8—H8	0.9300
O1—C10	1.240 (3)	C9—H9	0.9300
O3—C7	1.376 (3)	C2—H2	0.9300
O3—C1	1.412 (4)	C14—C15	1.429 (4)
C13—C11	1.383 (3)	C14—H14A	0.9700
C11—C12	1.459 (3)	C14—H14B	0.9700
C11—C10	1.466 (3)	C15—H15A	0.9700
C4—C3	1.366 (3)	C15—H15B	0.9700
C4—C5	1.376 (3)	C1—H1A	0.9600
C5—C6	1.371 (4)	C1—H1B	0.9600
C5—H5	0.9300	C1—H1C	0.9600
			
C13—S2—C15	95.41 (12)	O1—C10—C9	122.5 (2)
C13—S1—C14	96.14 (12)	O1—C10—C11	121.6 (2)
C8—N1—C12	121.5 (2)	C9—C10—C11	116.0 (2)
C8—N1—C4	119.61 (19)	C9—C8—N1	123.4 (2)
C12—N1—C4	118.88 (19)	C9—C8—H8	118.3
C7—O3—C1	117.8 (2)	N1—C8—H8	118.3
C11—C13—S1	121.55 (18)	C8—C9—C10	121.5 (2)
C11—C13—S2	123.24 (18)	C8—C9—H9	119.3
S1—C13—S2	115.21 (13)	C10—C9—H9	119.3
C13—C11—C12	118.8 (2)	C7—C2—C3	120.0 (2)
C13—C11—C10	120.5 (2)	C7—C2—H2	120.0
C12—C11—C10	120.8 (2)	C3—C2—H2	120.0
C3—C4—C5	120.0 (2)	C15—C14—S1	110.6 (2)
C3—C4—N1	119.8 (2)	C15—C14—H14A	109.5
C5—C4—N1	120.3 (2)	S1—C14—H14A	109.5
O2—C12—N1	119.6 (2)	C15—C14—H14B	109.5
O2—C12—C11	123.5 (2)	S1—C14—H14B	109.5
N1—C12—C11	116.9 (2)	H14A—C14—H14B	108.1
C6—C5—C4	119.6 (2)	C14—C15—S2	111.8 (2)
C6—C5—H5	120.2	C14—C15—H15A	109.3
C4—C5—H5	120.2	S2—C15—H15A	109.3
C5—C6—C7	120.6 (2)	C14—C15—H15B	109.3
C5—C6—H6	119.7	S2—C15—H15B	109.3
C7—C6—H6	119.7	H15A—C15—H15B	107.9
C4—C3—C2	120.6 (2)	O3—C1—H1A	109.5
C4—C3—H3	119.7	O3—C1—H1B	109.5
C2—C3—H3	119.7	H1A—C1—H1B	109.5
C2—C7—O3	125.1 (3)	O3—C1—H1C	109.5
C2—C7—C6	119.2 (2)	H1A—C1—H1C	109.5
O3—C7—C6	115.8 (2)	H1B—C1—H1C	109.5

### Hydrogen-bond geometry (Å, °)

**Table:**

*D*—H···*A*	*D*—H	H···*A*	*D*···*A*	*D*—H···*A*
C8—H8···O2^i^	0.93	2.42	3.259 (3)	150
C14—H14a···O1^ii^	0.97	2.685	3.475 (4)	139
C14—H14b···O1^iii^	0.97	2.709	3.513 (4)	141

Symmetry codes: (i) *x*+1/2, −*y*+1/2, *z*−1/2; (ii) *x*−1, *y*, *z*; (iii) −*x*+1, −*y*, −*z*+2.
